# Nonlinear thermo-optical properties of two-layered spherical system of gold nanoparticle core and water vapor shell during initial stage of shell expansion

**DOI:** 10.1186/1556-276X-6-448

**Published:** 2011-07-12

**Authors:** Victor K Pustovalov, Liudmila G Astafyeva

**Affiliations:** 1 Belarusian National Technical University, Independence pr. 65, Minsk, 220013, Belarus; 2 B.I. Stepanov Institute of Physics, National Academy of Sciences of Belarus, Independence pr. 68, 220072, Minsk, Belarus

## Abstract

Nonlinear thermo-optical properties of two-layered spherical system of gold nanoparticle core and water vapor shell, created under laser heating of nanoparticle in water, were theoretically investigated. Vapor shell expansion leads to decreasing up to one to two orders of magnitude in comparison with initial values of scattering and extinction of the radiation with wavelengths 532 and 633 nm by system while shell radius is increased up to value of about two radii of nanoparticle. Subsequent increasing of shell radius more than two radii of nanoparticle leads to rise of scattering and extinction properties of system over initial values. The significant decrease of radiation scattering and extinction by system of nanoparticle-vapor shell can be used for experimental detection of the energy threshold of vapor shell formation and investigation of the first stages of its expansion.

PACS: 42.62.BE. 78.67. BF

## Background

Metal nanoparticles (NPs) and other nanostructures are widely used in nanotechnology, physical chemistry, catalysis, biology, and laser nanomedicine for different purposes during past 10 years [1-15] (also see the references in these papers). The determination of sizes, concentrations, and placements of NPs in different media is carried out by different methods-transmission electron microscopy, small-angle X-ray scattering, laser scanning microscopy, optical diagnostics, etc. [1-15]. In many cases, optical detection and diagnostics of NPs via scattering have the advantages in comparison with others and can be carried out on the base of detection of radiation scattered from NPs placed in some medium (liquid). However, as NP radius *r*_0 _decreases, the scattered intensity drops as *r*_0_^6 ^and, as result, the detection difficulties will be increased [16]. Effective strategy for solving of this difficulty could be the use of nonlinear thermo-optical effects as a result of NP optical absorption and heating.

Nonlinear thermo-optical effects can be achieved under the action of laser radiation on NP, absorption of laser energy, NP heating, heat exchange with surrounding liquid, and its explosive vaporization. The liquid evaporates around rapidly heated NP, and spherical vapor shell (bubble) is formed near to an NP surface. It is possible to determine the temperature of heated NP, or determine the thermal refractive index change of ambient medium or formation of vapor shell (bubble) in the heated vicinity of absorbing NP [8,17]. The formation and expansion of vapor nanobubbles is attractive tool for diagnostics and applications in laser nanotechnology [8,18-26]. The resulting shell around laser-heated NP can cause spatial confined and highly localized thermomechanical damage to the surrounding medium. This feature should be taken into account for practical applications. The process is observed by means of the detection of transmission and scattering of the probe laser beam with wavelength 633 nm [19,22-26].

Nonlinear thermo-optical properties of two-layered spherical system of gold nanoparticle core and water vapor shell, created under laser heating of nanoparticle in water, were theoretically investigated in this paper.

## Results and discussion

At fairly short pulses of intense radiation, heating of NP and surrounding liquid medium (water) can occur at the rates of 10^12 ^to 10^14 ^K/s and more. Intense heat exchange in the surface layer of the NP drops the surface temperature of the NP and raises the temperature of the surrounding layers of water to a value of the order of the explosive ebullition (boiling) temperature of water [27] and higher. The nucleus vapor bubble originated in overheated water around the particle with achievement of threshold temperature in the range of 373 to 647 K (critical temperature for water) [27]. Thereafter, a very rapid (explosive) ebullition of the water occurs and the system goes into an equilibrium state characterized by the generation of a new phase-water vapor. A vapor shell formed around NP has an initial saturated water vapor pressure of about approximately 1 to10^2 ^atm at temperature of 100 to 500 C which induces a subsequent rapid expansion of the vapor shell [8].

Experimental investigations of vapor shell (bubble) formation and its dynamics in water under action of laser pulses on NP were carried out in [18-26]. It used gold NPs with diameters10 to 250 nm [18,19], 9 to 100 nm [20,21], 250 nm [22,23], and 30 nm [24-26] under laser pulse action with wavelengths *λ *= 400 nm [20,21], 532 and 527 nm [22-26], and 900 and 1,064 nm [18,19]. Continuous probe laser (*λ *= 633 nm) was used for monitoring of optical transmission through NP-shell area and for diagnostics of optical properties of NPs with surrounding vapor shells. Small-angle scattering method of the X-ray pulses was used for investigation of bubble properties in [20,21]. Formation and expansion of vapor bubble with radii of about 3*r*_0 _and more led to decreasing of transmission of probe laser radiation in mentioned above experiments. Experimental investigations of initial stage of vapor shell expansion did not carry out.

Nonlinear thermo-optical properties of two-layered spherical system of gold nanoparticle core and water vapor shell arising under laser heating of nanoparticle in water are theoretically investigated in this paper. The basic attention was given to the research of initial and following stages of bubble expansion. The investigation was performed on the base of theoretical modeling of absorption, scattering, and extinction of laser radiation with wavelengths *λ *= 532, 633, and 780 nm by system of NP core-vapor shell. It was assumed that the two-layered spherical system consists of a spherical homogeneous core of radius *r*_0 _with the complex refractive index *m*_0 _*= n*_0_-*iκ*_0 _of core material (gold), enveloped by the spherically symmetric homogeneous shell of radius *r*_1 _with the complex refractive index *m*_1 _*= n*_1_-*iκ*_1 _of water vapor shell. The particle is located in the homogeneous non-absorbing medium with a refractive index *n_m _*(water). Absorption , scattering , and extinction  efficiency cross sections were numerically calculated, where *K*_abs_, *K*_sca_, and *K*_ext _are efficiency factors of absorption, scattering, and extinction of radiation [16] by spherical system NP-core and vapor-shell (bubble) with outer shell radius *r*_1_.

Refractive index *m*_1 _of water vapor is presented in [28] in the ranges of radiation wavelengths 404 to 706 nm, temperature 100°C to 500°C and pressure 1 to 200 bar of water vapor. Analysis of presented values of *m*_1 _shows that the change of refractive index of water vapor *m*_1 _in the interval of wavelengths 404 to 780 nm on refractive index *m*_1 _of water vapor is equal to approximately 0.01% to 0.04% and can be neglected. For computer modeling of optical properties of shell, we choose one average value *m*_1 _≈ 1.001-*i*0. This value of refraction index of water vapor refraction *m*_1 _[28] can be used in the ranges of pressure approximately 5 to 20 bar and temperatures approximately 100°C to 500°C with deviation approximately 1%. Such parameters of water vapor are realized in experiments in real situation of formation and dynamics of bubble under action of moderate intensity (energy density) of laser pulses. Investigations of optical properties of pure gas (vapor) bubbles were carried out in [29]. Optical parameters of gold were taken from [30] and for surrounding water from [31].

**Figure 1:**
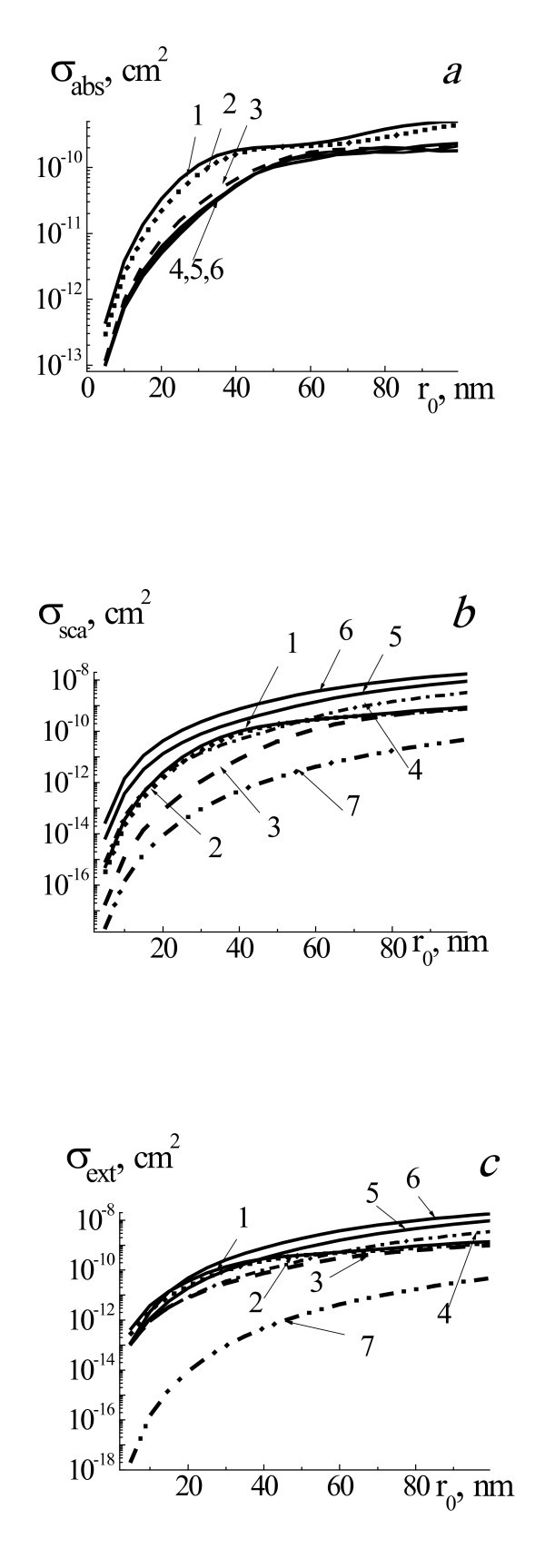
**Efficiency cross sections of absorption *σ*_abs_, scattering *σ*_sca_, and extinction *σ*_ext _of laser radiation**. Efficiency cross sections of absorption *σ*_abs _(**a**), scattering *σ*_sca _(**b**), and extinction *σ*_ext _(**c**) of laser radiation with wavelength 532 nm by two-layered spherical system gold NP core and water vapor shell placed in water for the range of NP radii *r*_0 _= 5 to 100 nm and radii of system *r*_1 _*= r*_0 _(1, pure gold NP, straight line) *r*_1 _*= r*_0 _*+ *0.1*r*_0 _(2, dotted line), *r*_1 _*= r_0 _+ *1*r_0 _*(3, dashed line), *r_1 _= r*_0 _*+ 2r_0 _*(4, dashed-dotted line), *r*_1 _*= r*_0 _*+ *3*r*_0 _(5, straight line), and *r*_1 _*= r*_0 _*+ *4*r*_0 _(6, straight line), and for homogeneous vapor bubble with *r*_0 _(7, dashed-double dotted line).

**Figure 2:**
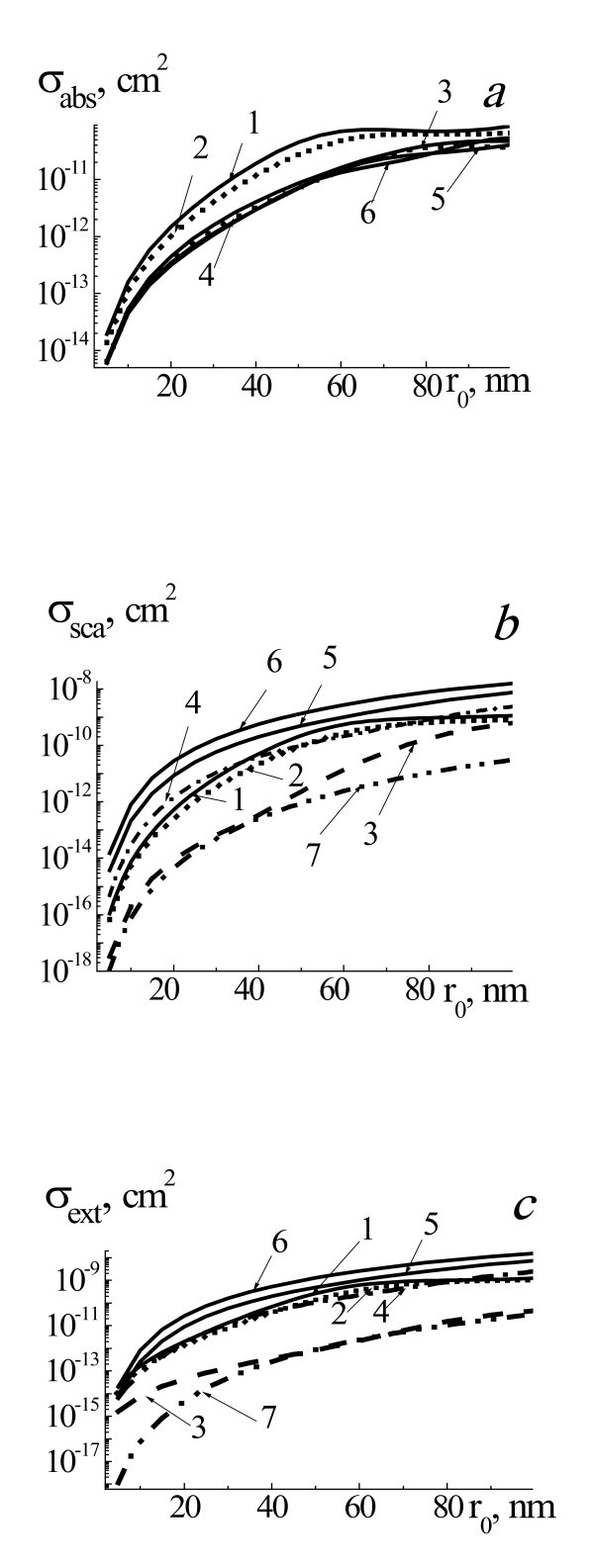
**Efficiency cross sections of *σ*_abs _(a), *σ*_sca _(b), and *σ*_ext _(c) of laser radiation**. With wavelength 633 nm by two-layered spherical system gold NP-core, water vapor-shell placed in water for the range of NP radii *r*_0 _= 5 to 100 nm, and radii of system *r*_1 _*= r*_0 _(1, pure gold NP, straight line), *r*_1 _*= r*_0 _*+ *0.1*r*_0 _(2, dotted line), *r*_1 _*= r*_0 _*+ *1*r*_0 _(3, dashed line), *r*_1 _*= r_0 _+*2*r*_0 _(4, dashed-dotted line), *r*_1 _*= r*_0 _*+ *3*r*_0 _(5, straight line), and *r*_1 _*= r*_0 _*+ *4*r*_0 _(6, straight line), and for homogeneous vapor bubble with *r*_0 _(7, dashed-double dotted line).

Figure 1 presents efficiency cross sections of absorption *σ*_abs_, scattering *σ*_sca_, and extinction *σ*_ext _of laser radiation with wavelength 532 nm by two-layered spherical system gold NP core and water vapor shell, placed in water for the range of NP radii *r*_0 _= 5 to 100 nm and radii of system *r*_1 _*= r*_0 _(pure gold NP), *r*_1 _*= r*_0 _*+ *0.1*r*_0_, *r*_1 _*= r*_0 _*+ *1*r*_0_, *r*_1 _*= r*_0 _*+ *2*r*_0_, *r*_1 _*= r*_0 _*+ *3*r*_0_, and *r*_1 _*= r*_0 _*+ *4*r*_0_, and for homogeneous vapor bubble with radius *r*_0 _= 5 to 100 nm. Increasing of vapor shell thickness leads to substantial monotonous decreasing of *σ*_abs _from two to eight times for all range of 5 <*r*_0 _< 100 nm and for the interval of shell vapor thicknesses Δ*r*_1 _<*r*_0_, Δ*r*_1 _= *r*_1_-*r*_0_. Further increasing of vapor shell thickness weakly influences the *σ*_abs_. It means that the absorbance of laser radiation by the NP is sharply decreased at formation of thin shielded vapor shell and then it is weakly changed for thick shells. Cross section of absorption *σ*_abs _for pure vapor bubble is much smaller than *σ*_abs _for system NP-shell and does not present at Figures 1 and 2.

Even appearance of vapor shells with thickness Δ*r*_1 _*≤ r*_0 _leads to decrease of *σ*_sca _from 10 to 30 times in the NP radius interval 5 <*r*_0 _< 50 nm. When Δ*r*_1 _becomes vastly larger than *r*_0 _(Δ*r_1 _≈ *4*r*_0_), values of *σ*_sca _grow from 10 to 50 times for all values of *r*_0 _in comparison with initial value *σ*_sca _for pure NP. As to *σ*_ext_, the dependences of the extinction of laser radiation of such system NP core-water vapor shell on *r*_0 _and vapor shell thickness Δ*r*_1 _resemble that in the case of scattering. Values of *σ*_ext _decrease for all values of *r*_0 _in the interval of vapor shell thicknesses Δ*r*_1 _*≤ r*_0 _and then grow at first for large values of core radii and thereafter in all intervals of the core sizes. We see nonlinear dependence of *σ*_sca _and *σ*_ext _on vapor shell thickness during bubble formation, and increase of Δ*r*_1 _till Δ*r*_1 _*≤ r*_0 _leads to significant decrease of *σ*_sca _and *σ*_ext_. Following increase of Δ*r*_1 _*> r*_0 _leads to increase of *σ*_sca _and *σ*_ext_.

Figure 2 presents efficiency cross sections of absorption *σ*_abs_, scattering *σ*_sca_, and extinction *σ*_ext _of probe laser radiation with wavelength 633 nm by two-layered spherical system gold NP core and water vapor shell for the range of NP radii *r*_0 _= 5 to 100 nm and different radii of system and for homogeneous vapor bubble with *r*_0_. Influence of vapor shell thickness on thermo-optical properties of system NP-vapor shell for probe radiation with wavelength 633 nm is analogical one as for the case of laser radiation with wavelength 532 nm. It is especially extended to the cross sections of absorption *σ*_abs _of considered NPs. In the case of the cross sections of scattering and extinction character of dependences *σ*_sca_(*r*_0_) and *σ*_ext_(*r*_0_) for different values of Δ*r*_1 _are similar. Furthermore, when increasing Δ*r*_1 _(Δ*r*_1 _*≈ *4*r*_0_), values of *σ*_sca _grow from 100 to 10 times in the dependence on the *r*_0_. Notice that the scattering and extinction cross sections of homogeneous water vapor bubbles of different sizes in water are very small and is 2-3 orders less than for pure gold and two-layered system NP-vapor shell (line 7, Figures 1 and 2).

It is well known that the formation of vapor bubble in liquid leads to significant increasing of radiation scattering, and extinction by bubble and bubble itself can be visible [16,32]. Nonlinear behavior mentioned above (decreasing of *σ*_abs_, *σ*_sca_, and *σ*_ext _during increasing of Δ*r*_1 _till Δ*r*_1 _*≤ r*_0_) leads to bleaching of medium during initial stage of vapor shell expansion. This behavior exists for different values of *m*_1_.

**Figure 3:**
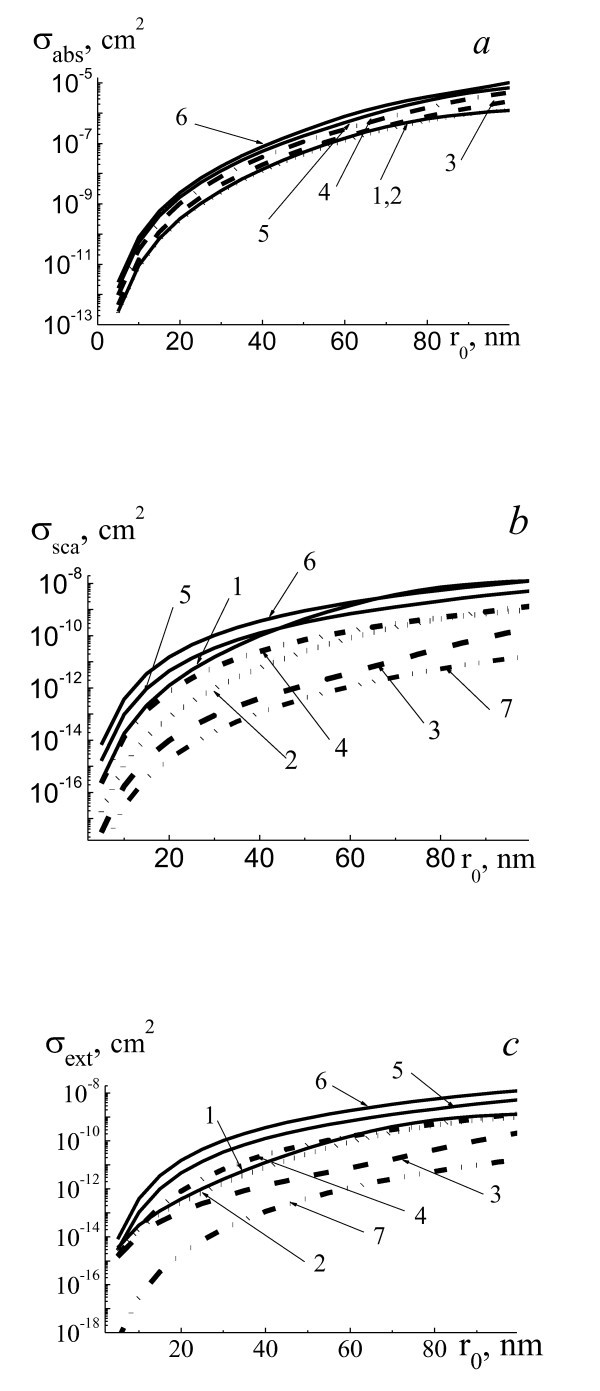
**Efficiency cross sections *σ*_abs _(a), *σ*_sca _(b), and *σ*_ext _(c) of laser radiation**. With wavelength 780 nm by two-layered spherical system gold NP core and water vapor shell with refractive index of vapor *m*_1 _*= *1.001-*i*0 placed in water for the range of NP radii *r*_0 _= 5 to 100 nm and radii of system *r*_1 _*= r*_0 _(1, pure gold NP, straight line), *r*_1 _*= r*_0 _*+ *0.1*r*_0 _(2, dotted line), *r*_1 _*= r*_0 _*+ *1*r*_0 _(3, dashed line), *r*_1 _*= r*_0 _*+ 2r*_0 _(4, dashed-dotted line), *r*_1 _*= r*_0 _*+ *3*r*_0 _(5, straight line), and *r*_1 _*= r*_0 _*+ *4*r*_0 _(6, straight line), and for homogeneous vapor bubble with *r*_0 _(7, dashed-double dotted line).

Figure 3 presents efficiency cross sections of absorption *σ*_abs_, scattering *σ*_sca_, and extinction *σ*_ext _of laser radiation with wavelength 780 nm by two-layered spherical system gold NP core and water vapor shell with refractive index of vapor *m*_1 _= 1,001-*i*0. The increase of vapor shell thickness till Δ*r*_1 _<*r*_0 _for *λ *= 780 nm leads to insignificant decrease of efficiency cross sections of absorption *σ*_abs _(10% ÷ 15%), and then, at increase in a shell thickness to five times, absorption grows almost in 10 times (Figure 3a). In the case of scattering and extinction of NPs (Figure 3b,c), the dependencies of *σ*_sca_(*r*_0_) and *σ*_ext_(*r*_0_) for wavelength 780 nm are similar as for other considered wavelengths.

**Figure 4:**
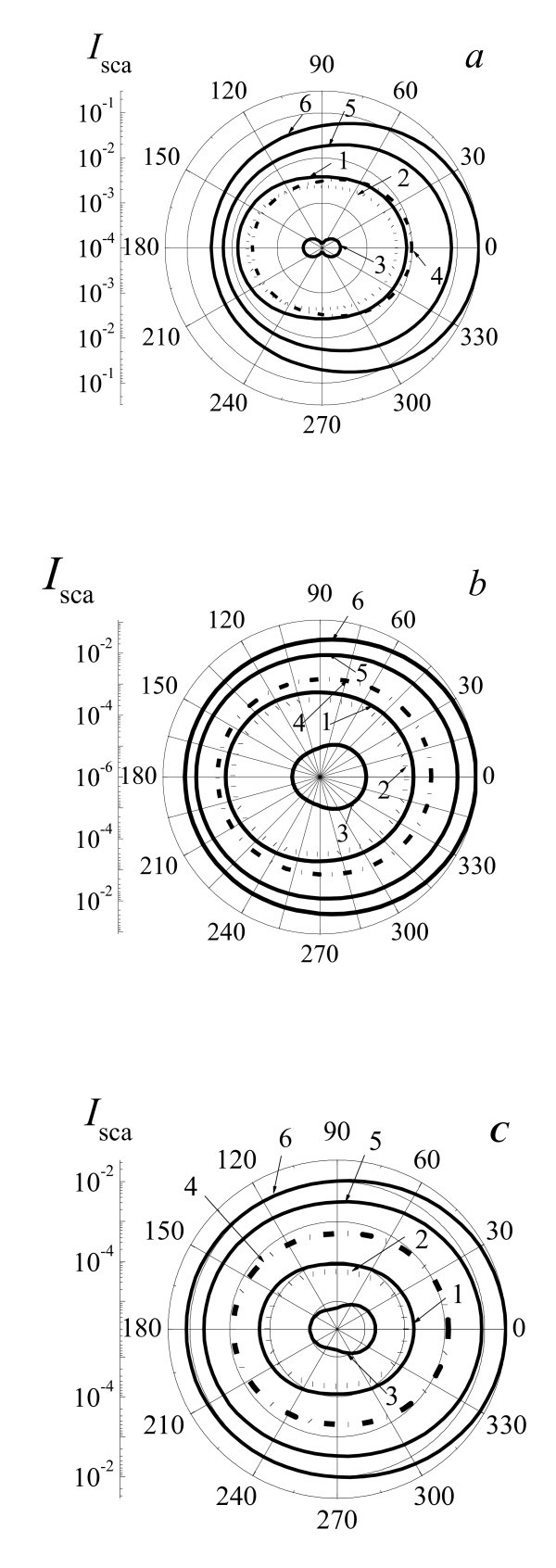
**Angular distributions (optical indicatrixes) of radiation intensity *I*_sca_**. With wavelength 532 nm (**a**), 633 nm (**b**), and 780 (**c**) nm scattered by two-layered spherical system gold NP core and water vapor shell placed in water for the NP radius *r*_0 _= 20 nm and radii of system *r*_1 _*= r*_0 _(1, pure gold NP, straight line), *r*_1 _*= r*_0 _*+ *0.1*r*_0 _(2, dotted line), *r*_1 _*= r_0 _+ *1*r*_0 _(3, straight line), *r*_1 _*= r*_0 _*+ *2*r*_0 _(4, dashed-dotted line), *r*_1 _*= r*_0 _*+ *3*r*_0 _(5, straight line), *r*_1 _*= r*_0 _*+ *4*r*_0 _(6, straight line). Direction of laser radiation propagation is from left to right (from 180° to 0°). Polar coordinates show angles for scattered radiation in the range 0° to 360°; scale *I*_sca _shows arbitrary units of intensity.

Figure 4 presents angular distributions (optical indicatrixes) of radiation intensity *I*_sca _with wavelengths λ = 532, 633, and 780 nm scattered by two-layered spherical system gold NP core and water vapor shell for the NP radius *r*_0 _= 20 nm and different radii of system *r*_1_. The increase of vapor shell thickness till Δ*r*_1 _≤ *r*_0 _for *λ *= 532, 633, and 780 nm leads to decrease of scattered radiation intensity in approximately 50 ÷ 300 times in all scattered directions. Only at Δ*r*_1 _≈ 2*r*_0 _scattering intensity is approximately equal initial distribution of scattered radiation from pure NP. Then, further growth of vapor shell thickness tends to essential increase of scattered radiation intensity (in 20 ÷ 100 times for Δ*r*_1 _= 4*r*_0_) in comparison to the case of pure Au NP. This fact is well correlated with the behavior of *σ*_sca _(Figures 1 and 2). With growth of Δ*r*_1_, optical indicatrixes become essentially extended in the forward direction (angle 0°). We have to note that mathematical modeling of optical indicatrixes of scattered radiation was independently carried out on the base of optical constants without use of calculated values of *σ*_sca_. This behavior of indicatrixes of scattered radiation is additional evidence of nonlinear (decreasing and increasing) properties of system NP core and vapor shell during initial stages of bubble expansion till *r*_1 _≤ 2*r*_0_.

**Figure 5:**
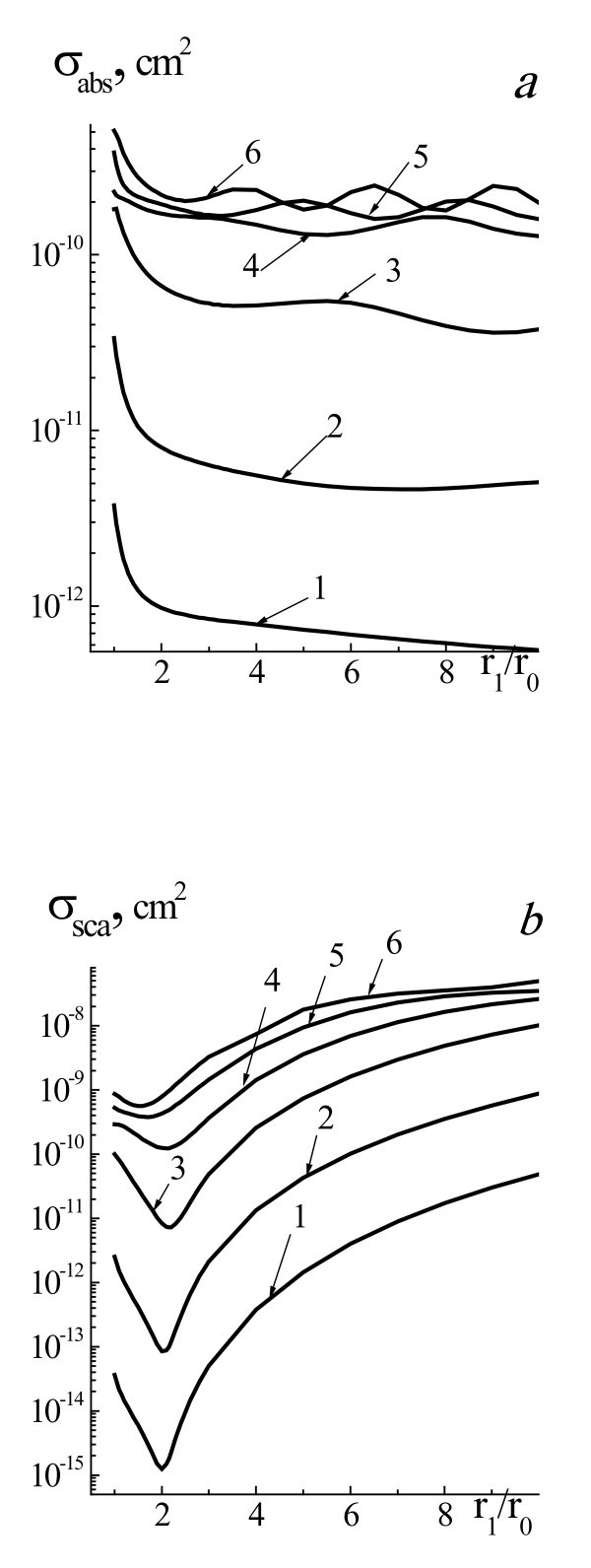
**Efficiency cross sections *σ*_abs _(a) and *σ*_sca _(b) of laser radiation**. With wavelength 532 nm by two-layered spherical system gold NP core and water vapor shell for the radii *r*_0 _= 10 nm (1), 20 nm (2), 40 nm (3), 60 nm (4), 80 nm (5), and 100 nm (6), and for the range of system radii *r*_1 _= (1 ÷ 10) *r*_0_.

Figure 5 presents cross sections *σ*_abs _and *σ*_sca _of laser radiation with wavelength 532 nm by two-layered spherical system gold NP core and water vapor shell for the radii *r*_0 _= 10, 20, 40, 60, 80, and 100 nm as a function of the radii relations *r*_1_/*r*_0 _in the interval of radii *r*_1 _= (1 to 10)*r*_0_. As shown in Figure 4a, absorption cross sections *σ*_abs _are decreased for all range of 1 <*r*_1_/*r*_0 _< 10 and for the interval of core radii 10 nm ≤ *r*_0 _≤ 100 nm. At first, this decrease is reasonably sharp from two to four times, and then after achievement of value Δ*r*_1 _≤ *r*_0_, *σ*_abs _slowly reduces. It is determined by the shielded effect of vapor shell when irradiation cannot reach the absorbing core. The growth of the core radii results in essential increase of absorption cross sections *σ*_abs _as long as *r*_0 _≤ 60 nm. For *r*_0 _≤ 60 nm, the dependence of *σ*_abs_(*r*_0_) becomes oscillating and undergoes less effect of core radius. Scattering cross sections of *σ*_sca _are also lowered in the interval of 1 <*r*_1_/*r*_0 _< 2 (Figure 4b). Then, *σ*_sca _is sharply increased in the interval *r*_1_/*r*_0 _= 2 to 10. Scattering cross section *σ*_sca _is decreased up to one to two orders of magnitude depending on *r*_0_, for example, for *r*_0 _= 20 nm is decreased from σ_sca _= 2.5 × 10^-12 ^cm^2 ^(*r*_1_/*r*_0 _= 1) to *σ*_sca _= 8.5 × 10^-14 ^cm^2 ^(*r*_1_/*r*_0 _= 2) for *λ *= 532 nm. After achievement of minimal value, *σ*_sca _increases and at *r*_1 _= (2 to 3.5) *r*_0 _cross section *σ*_sca _achieves initial value of *σ*_sca_(*r*_1 _= *r*_0_). It means that the scattering property of system NP-vapor shell is equal initial value of *σ*_sca _for pure NP at this value of *r*_1_. After this moment, the increase of *r*_1_/*r*_0 _leads to growth of *σ*_sca _up to values two to four orders of magnitude greater than initial values of this one. This effect is due to the complicated two-layered spherical system, and the fact that the growth of vapor shell leads to change of median complex refractive index of two-layered NP: the real part of the complex refractive index increases and the imaginary part is decreased. Therefore, at first, the scattering cross sections of NPs fall and then begin to grow when expanding the vapor shell thickness and value of *r*_1_/*r*_0 _increases.

**Figure 6:**
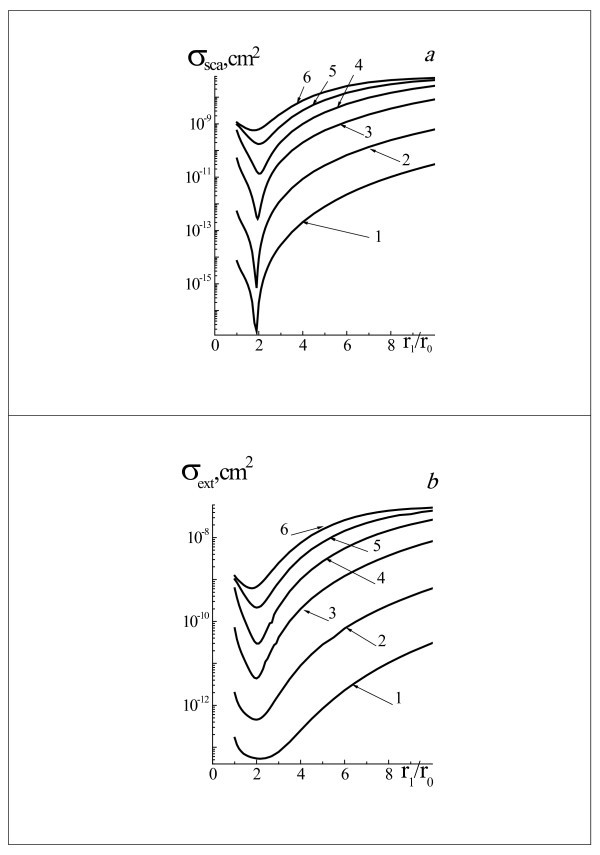
**Efficiency cross sections *σ*_sca _(a) and *σ*_ext _(b) of laser radiation**. With wavelength 633 nm by two-layered spherical system gold NP core and water vapor shell for the radii *r*_0 _= 10 nm (1), 20 nm (2), 40 nm (3), 60 nm (4), 80 nm (5), and 100 nm (6), and for the range of system radii *r*_1 _= (1 ÷ 10) *r*_0_.

Figure 6 shows the scattering *σ*_sca _and extinction *σ*_ext _cross sections of probe laser radiation with wavelength 633 nm by two-layered spherical system gold NP core and water vapor shell for the radii *r*_0 _= 10, 20, 40, 60, 80, and 100 nm as a function of the radii relations *r*_1_/*r*_0 _in the interval of radii *r*_1 _= (1 to 10) *r*_0_. Influence of vapor shell thickness on scattering properties of system NP-vapor shell for probe radiation with wavelength 633 nm is analogical as for the laser radiation with wavelength 532 nm. Character of dependences *σ*_sca_(*r*_0_) and *σ*_ext_(*r*_0_) for different values of *r*_0 _are similar. Extinction cross section *σ*_ext _is decreased from 2 to 20 times depending on *r*_0_, for example, for *r*_0 _= 40 nm is decreased from *σ*_ext _= 6.2 × 10^-10 ^cm^2 ^(*r*_1_/*r*_0 _= 1) to *σ*_ext _= 3 × 10^-11 ^cm^2 ^(*r*_1_/*r*_0 _= 2) for *λ *= 633 nm.

## Conclusions

We found the general trends of nonlinear behavior of NP-vapor shell system-decrease of absorption and decrease and subsequent increase of scattering and extinction with increasing of shell radius, beginning from the initial period of shell expansion. Vapor shell formation can produce one to two orders of magnitude of decreasing of scattered radiation during initial stage of shell expansion till radius *r*_1 _≤ 2*r*_0_. The amplification of scattering intensity is mainly due to increasing of shell radius *r*_1 _*>*2*r*_0_.

Such behavior of thermo-optical properties of spherical system gold NP core and water vapor shell depending of shell thickness Δ*r*_1_, NP radius *r*_0_, wavelength, and optical properties of vapor (pressure and temperature of vapor) can open new options for optical detection of the moment of vapor shell formation and investigation of the initial stage of its dynamics with small thickness of vapor shell.

Different situations can be realized. Optical detection of single NP is usually realized by irradiation of probe laser radiation and optical detection of scattered radiation and extinction by NP. Suppose that single NP can be visualized using of probe radiation without laser pump irradiation and vapor shell formation, it means that optical scattering of radiation by pure single NP is enough to be detected. After laser pump irradiation and shell formation and during initial stage of shell dynamics with Δ*r*_1 _≤ *r*_0_, intensity of scattered radiation by system NP-shell will be decreased (see Figures 1, 2, 3 and 4), and this system could not be visualized. Only after substantial increasing of Δ*r*_1 _up to Δ*r*_1 _≈ (2 to 3) *r*_0 _and more and increasing of intensity of scattered radiation by system NP-shell it will be possible to visualize this system.

Optical detection of system of NPs in some medium is based on the detection of transmitted radiation through this dispersed medium. The formation of vapor shells with small thicknesses on NPs under pump laser irradiation leads to substantial decreasing of *σ*_ext _for probe radiation 633 nm (see Figures 1, 2, and 3). It means that the moment of initial formation of nanoshells around NPs can be detected by increasing of transmitted probe radiation intensity. Then, after substantial increasing of Δ*r*_1 _>*r*_0 _up to Δ*r*_1 _≈ (2 to 5) *r*_0 _transmitted probe intensity will be decreased.

Applications of laser-induced vapor nanoshells are proposed for selective tissue damage on the cellular level, anticancer therapy, when selective destruction of cells containing NPs can be triggered due to these ones [3-8]. Vapor nanoshells formed around laser-heated NPs can serve as contrast agents in optical diagnostics and optoacoustic tomography, etc. Vapor bubble formation around NPs and its expansion can be used for optical limiting and switching in suspensions.

The significant decrease of radiation scattering and extinction by system of nanoparticle-vapor shell can be used for experimental detection of the energy threshold of bubble formation and investigation of the first stages of its expansion.

## Methods

We used modified Mie theory developed for two-layer spherical system particle-shell [33,32,35] to model absorption, scattering, and extinction of pump *λ *= 532 and 780 nm and probe *λ *= 633 nm radiations by spherical system of gold NP core and water vapor shell. The expressions for the optical characteristics of two-layered sphere (efficiency cross sections of absorption, scattering, and extinction) are presented in terms of the amplitude coefficients given by the theory of diffraction of electromagnetic radiation on two-layered spherical particle [33,32,35].

## Abbreviations

NPs: nanoparticles.

## Competing interests

The authors declare that they have no competing interests.

## Authors' contributions

VKP and LGA carried out all investigations together. All authors read and approved the final manuscript.

## Authors' information

VKP is a professor of Belarusian National Technical University, Independence pr. 65, Minsk, 220013, Belarus. LGA is a chief scientist of B.I. Stepanov Institute of Physics, National Academy of Sciences of Belarus, Independence pr. 68, 220072, Minsk, Belarus.
